# Effect of Rural Clinical Placements on Intention to Practice and Employment in Rural Australia: A Systematic Review

**DOI:** 10.3390/ijerph19095363

**Published:** 2022-04-28

**Authors:** Claire Ellen Seaman, Elyce Green, Kate Freire

**Affiliations:** Three Rivers Department of Rural Health, Charles Sturt University, Wagga Wagga, NSW 2678, Australia; elgreen@csu.edu.au (E.G.); kfreire@csu.edu.au (K.F.)

**Keywords:** higher education policy, health education, clinical placements, rural training

## Abstract

Background: Supporting the provision of clinical placement (CP) experiences in rural areas is a strategy used worldwide to promote the rural health workforce. While there is international evidence for this intervention in medicine, there is limited understanding of the influence of rural CP for nursing, midwifery, allied health, and dentistry health professions in Australia, which have received substantial federal investment. This review examined the relationship between rural CP and non-medicine health students’ future rural practice intentions and workforce outcomes. Methods: Four databases were systematically searched; papers were screened using defined criteria and appraised using the mixed-methods appraisal tool (MMAT). Findings were synthesized using a critical narrative approach. Results: The methodological quality of the 29 eligible studies (13 quantitative non-randomized, 10 mixed method, 4 qualitative, 2 quantitative description) was appraised. Ten high-quality studies were identified. The review found that positive CP experiences may influence intention to practice rurally amongst undecided students and serve as a reinforcing experience for those students already interested in rural practice. There were mixed findings regarding the influence of CP length. The review also found that there is currently only evidence for the short-term effects of CP on students’ future practice outcomes in rural areas with focus thus far on early practice outcomes. Conclusions: Those looking to use rural CP to promote the rural health workforce should focus on supporting the quality of a large number of CP experiences that are undertaken in rural areas, as there are currently differing findings on the role of rural CP length. Future studies of rural CP should consider greater use of social and educational theories to guide them.

## 1. Introduction

Health profession student clinical placement (CP) opportunities in rural communities are commonly integrated into tertiary education curricula to provide students with exposure to rural health skills and practice opportunities [1]. Rural CP experiences are also part of a global effort to recruit and retain health staff in rural locations [1] and are a central component of rural workforce strategy in Australia towards equitable health service delivery for rural people [2]. There is some evidence of this intervention positively contributing to the recruitment of medical students to the rural health workforce [3]; however, the WHO has previously highlighted the limited evidence worldwide of CP influence on rural practice interest or workforce outcomes for other health professionals [1]. More recently, a review of national policies to address rural workforce maldistribution in select OECD countries found Australia has produced the best evidence for recruitment impact from rural CP, likely due to national funding of rural CP [4], described below. This systematic review critically appraises the current body of evidence focused on the relationship between Australian rural CP undertaken by health professions students in disciplines of nursing, midwifery, dentistry, oral health, and allied health (all other tertiary-level health professions degrees), and their rural workforce intentions or employment. The terms ‘rurality’ and ‘CP’ both arouse different understandings and expectations, including across disciplines and nations. This multidisciplinary review therefore focuses only on rural CP undertaken in Australia as a leading national context for evidence of rural CP effects. 

For this study, CPs are defined as set blocks of time during tertiary study where health students experience training in a clinical, health, or other organisational settings (i.e., schools, community centres, government agencies) for the purpose of work-integrated learning. This excludes community immersions that do not involve practice-based learning. CP supports authentic engagement with industry to develop the occupational skills required as students transition towards becoming accredited practitioners [5,6]. CP experience also enable students to engage in a multitude of different practice communities and therefore to discover and assess possibilities for their future professional practice aligned with their developing professional identity [5,6]. 

Rural CPs are expected to support students to positively discover and assess the possibilities of rural health practice through meaningful engagement with associated communities [7]. In Australia, the federal government has supported rural CPs for nursing and midwifery, allied health, and dentistry for more than twenty years via University Departments of Rural Health (UDRHs) [2]. Since 2016, UDRHs have been distinctly funded under the same Rural Health Multidisciplinary Training (RHMT) Program as Rural Clinical Schools (RCS) which are specific to medicine, with investment in the RHMT Program costing approximately AUD 200 million per annum [7]. Key aims of UDRHs and RCSs include enhancing the future rural health workforce through providing health students with positive, longer-term CP experiences, as well as supporting the recruitment of students from rural areas into health professions degrees [7]. These aims are supported by substantial international and Australian evidence that students’ prior rural living is a predictor of rural workforce intention and uptake [1,3,8]. There is also some evidence from medicine that longer-term rural CP can increase the likelihood of future rural practice [3]. Despite the ongoing investment in UDRHs, the effectiveness of rural CP for attracting non-medicine health professionals to rural practice is not well understood [7,8].

Finally, there are calls for enhanced, meaningful engagement with educational and sociological theories in health education research, including studies of work-integrated learning experiences, to better inform health education strategies [9,10,11]. In rural health, health professions education, and workforce research, there is increasing use of place-based understandings and situated learning frameworks to critically interrogate the socio-political contexts of interventions and inform best practice [11,12,13,14]. The funding allocated to multidisciplinary rural CP in Australia suggests this could be a site for innovative pedagogical practices. Such innovations could inform rural workforce recruitment efforts in other nations, as well as broader health education practices through testing and developing relevant theories. It was therefore pertinent in this review to also assess the existing use of theory in studies of rural CP for allied health, dentistry, nursing, and midwifery students. 

The aim of this systematic review was to examine the research on non-medicine CP and rural practice intentions and rural workforce outcomes. The following research questions were used to guide the review:

1. What is the influence of rural CP on intention to practice rurally?
2. What is the influence of rural CP on future rural practice?
3. What theoretical frameworks are used in research to explore these relationships?

## 2. Materials and Methods

### 2.1. Search Strategy

A preliminary search of PROSPERO, the Cochrane Database of Systematic Reviews, and the JBI Database of Systematic Reviews and Implementation Reports was conducted, and no current or in-progress systematic reviews on the topic were identified. The review followed the JBI methodology for systematic reviews and the protocol was registered with PROSPERO (CRD42021235448). Database searches were conducted using EBSCOhost (health) (inclusive of CINAHL Plus), PubMed, Web of Science, and Scopus with combinations of keywords related to rural health CP and the outcomes of recruitment, retention, intention, workforce, employment, or career. An example of keyword combinations and database searches are shown in Appendix A. Citation tracking of included studies was also used to identify potential studies for inclusion in the review. Searches were first conducted in January 2020 and were repeated in January 2021 and April 2022.

### 2.2. Article Selection

All identified citations were uploaded into EndNote X8 and the duplicates removed. Titles and abstracts were screened by two independent reviewers against the inclusion/exclusion criteria set for this review (Table 1). 

Disagreements were resolved by a third reviewer. Full texts of potentially relevant articles were then assessed against the inclusion criteria by two independent reviewers. Disagreements were again resolved by a third reviewer. Reasons for exclusion of articles at full text were recorded using the Preferred Reporting Items for Systematic Reviews and Meta-Analyses (PRISMA) as a guide [15].

For this review, the definition of rurality was kept broad as there is a variety of approaches to rurality used in studies in this area [16]. Where information on the location of the rural CP is available, ‘rural’ is defined as outside of Australia’s ‘major city areas’ per the Australian Bureau of Statistics’ remoteness area classification [17]. Otherwise, the study meets the inclusion criterion if the study has defined placements as ‘rural’ inclusive of ‘regional’ and ‘remote’ terminology. 

### 2.3. Assessment of Methodological Quality

All included studies were critically appraised by two independent reviewers for methodological quality using a modified Mixed Methods Appraisal Tool (MMAT) [18]. The MMAT was chosen to assess methodological quality because it can facilitate examination of different methods. For this review, studies were allocated to a study type based on the approach described in-text. With this approach, one alteration to the MMAT was made with the fifth quality criterion for non-randomised quantitative studies—‘During the study period, was the intervention administered (or exposure occurred) as intended?’ replaced by the descriptive survey study criterion, ‘Is the statistical analysis appropriate to answer the research question?’. This substitution was made given the prevalence of survey-based studies, as well as the variation in ‘the intervention’ under study—the characteristics and contexts of multidisciplinary rural CP [7,8]. The reviewers agreed that appropriate statistical analysis is therefore a more relevant indicator of study quality than attempting to assess whether the intervention was administered as intended. Reviewer-agreed definitions of low-, medium-, and high-quality papers (meets 0–2, 3, and 4–5 criteria, respectively) were applied to the screening. Disagreements on researchers’ ratings were resolved by discussion amongst the three authors. In accordance with the MMAT, no article was excluded from the review based upon its score; however, review results place greater emphasis on the findings of studies from articles that were rated as having high methodological quality [18].

### 2.4. Data Extraction and Synthesis

Data from the included studies were extracted into a spreadsheet, including reported study design, research question or aim, student discipline, placement length, setting, rural origin, rural origin accounted for in analysis, analysis, rural practice intention outcomes, rural employment outcomes, and results. Two reviewers extracted data independently which was then combined into one dataset by a reviewer, with discrepancies resolved by discussion among the reviewers. Due to the heterogeneous nature of the study designs, data were synthesised by a critical narrative summary guided by the results of the quality screening. 

## 3. Results

### 3.1. Identification and Selection of Articles

**Figure 1:**
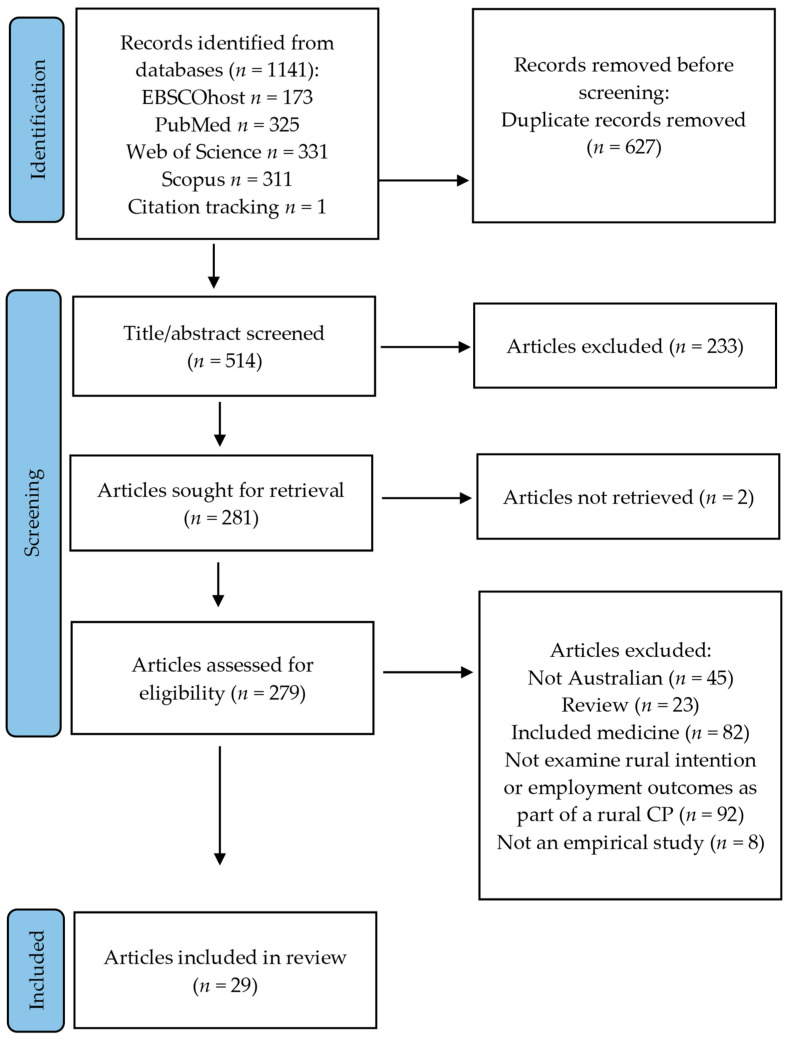
PRISMA flow chart of included articles, based on Page et al., 2021 [15].

The results of the search and the article inclusion process are reported in Figure 1. Database searching resulted in 514 potential papers once duplicates were removed, and 233 papers were excluded through abstract screening. Full-text retrieval and full-text screening resulted in the exclusion of a further 252 papers. Twenty-nine articles were included in the review.

### 3.2. Characteristics of Included Studies

Seven studies were published between 2000 and 2009, thirteen from 2010 to 2019, and nine from 2020 to 2021. The studies investigated students’ CP across several health professions, listed in Table 2. The most common rural CP duration was one month (*n* = 7, 24%). Where one CP experience was examined, CP duration ranged from one week to one year. Multiple CP experiences were examined cumulatively as total weeks or total number of CP. Five studies (17%) did not report the duration of the CP under examination. 

### 3.3. Methodological Quality 

The included reports used four different study designs: quantitative non-randomised (*n* = 13), mixed method (*n* = 10), qualitative (*n* = 4), and quantitative descriptive (*n* = 2) (Table 3). Nearly half of the studies were assessed as low quality (*n* = 14, 48%), with five (17%) assessed as medium quality, and ten (34%) as high-quality studies. All four qualitative studies were rated as low (*n* = 1) to medium quality (*n* = 3). The 13 studies that employed a non-randomised quantitative design were largely rated as high quality (low *n* = 3, medium *n* = 1, high *n* = 9). The 10 studies that used a mixed method study design were evaluated as predominately low quality (low *n* = 8, medium *n* = 1, high *n* = 1). Five papers were found to have met the criterion regarding the rationale for using mixed methods design; however, no papers met the criterion adhering to the quality criteria of each tradition of the methods. There was a higher rate of medium- or high-quality papers that examined rural practice (*n* = 10, 63%) than rural intention (*n* = 6, 40%) outcomes. In conclusion, critical appraisal found the overall methodological quality of the included papers was mixed.

### 3.4. Review Findings

A summary of the review findings is found in Table 4.

#### 3.4.1. Rural CP Influence on Intentions and Attitudes towards Rural Practice

Fifteen studies examined the relationship between students’ rural CP experiences and their intentions and attitudes towards future rural practice, including two that also examined employment. All six medium–high-quality papers considered the role of rural background or existing rural interest on rural practice intentions or attitudes [25,36,42,44,45,47]. Five of these studies relied on cross-sectional post-placement evaluation, with only one [42] using longitudinal, pre-post evaluation. Synthesis of the studies showed mixed findings on the relationship between rural CP and interest in future rural practice. There was no significant difference in practice intention associated with different placement durations in one multidisciplinary study [25], while another found all placement lengths were associated with encouraging consideration of rural practice [47].

Study results point to the importance of prior rural living, experience, or interest when assessing placement impact on intentions or attitudes towards rural practice. Among pharmacy students, rural CPs were significantly associated with increased likelihood of intending to practice rurally in students’ final year relative to their first year [42]; however, this only approached significance when controlling for the positive effect of rural background [42] (p. 308). A study of allied health students [47] found that rural CPs were only significantly associated with increased intention to practice rurally among city-background students. However, the survey item asked whether the rural CP made students ‘reconsider their future’ towards rural practice, and there was already high pre-placement interest in rural practice among rural background students. Similar findings were reported in studies of nursing students [26] and dentistry students [20,29,30]. Johnson and Blinkhorn [29,30] also found a rural CP experience positively encouraged ‘undecided’ dental students towards interest in rural practice. Somewhat distinctly, a multidisciplinary study of whether the CP ‘encouraged’ consideration of rural practice found that only placement quality (satisfaction with educational resources and CP overall) and prior interest in working rurally had a significant and positive association, while rural background and differing placement lengths did not [25]. A study where intention change was qualitatively examined distinctly for city and rural background students also found evidence that a rural CP more positively affected the intention of city-background students [45]. 

In more remote settings, a study found that quality rural CP can highlight the professional benefits of rural practice and develop students’ cultural capabilities working with First Nations peoples to encourage work in this area [44,45]. Despite this, there was low interest in long-term remote practice, attributed to feelings of social isolation [44]. Finally, rural CPs may also serve to discourage students from rural practice. For instance, a study of final year nurses found rural CPs gave students a better understanding of what their graduate year might look like in a rural hospital, including being dissuaded from rural practice by demanding workloads and lack of graduate support [37]. 

Overall, this indicates that positive CP experiences may influence intention most among those for whom rural practice has not previously been a major consideration, as those from a rural background or already highly interested in rural practice may seek out rural placement opportunities. Concrete findings are limited by variation of intention measures across studies, as well as the very limited statistical or theory-informed consideration of item reliability and validity.

#### 3.4.2. Rural CP Influence on Rural Practice 

Sixteen papers examined the effect of students’ rural CP experiences on their subsequent working location, including two [19,44] that also examined rural intention. Ten of the papers were rated as medium or high quality, eight of which considered the effect of rural background on rural practice [24,28,33,38,39,40,41,44]. This review found some evidence that quality placements are positively associated with early practice outcomes; however, evidence for long-term effects was limited and findings more broadly were limited by the self-selection of students into rural training experiences. 

It is not possible to reach a clear conclusion from this review on the effect of rural CP length on rural practice. Contrary to policy expectations, Playford et al. (2006) [38] found longer rural CP duration (more than one month) to be negatively associated with rural practice compared to CP of less than one month, controlling for rural background. Volunteering for the CP and perceived excellence in professional development gained through the CP was also significantly and positively associated with rural practice. The authors suggest that shorter lengths of CP may contribute to metropolitan and other travelling students’ positive experience through reducing financial burden and social dislocation when in accommodation away from paid work and family and friends. Another study attempted to control for this by asking questions about barriers to undertaking CP, although it is not clear whether these were rural CP-specific [46]. In this study, propensity score matched analysis of survey data from multidisciplinary health professionals 1–14 years after graduation found that professionals in the highest quintile of cumulative placement length worked significantly more hours rurally [46].

Quality-rated studies have also found some evidence that rural CP length is positively associated with future rural practice, using data from the recently developed, multi-institutional Nursing and Allied Health Graduate Outcome Tracking (NAHGOT) study [48]. NAHGOT is a longitudinal study that is currently in its early stages, but which aims to comprehensively track individuals’ health professional education, CP, and practice journey through linking institutional and professional registration data [48]. 

Using university data, two NAHGOT papers each computed a polytomous variable of total time spent on rural CP across students’ degree [28,41]. Both found that likelihood of rural practice increased the time a student spent in rural CP; however, this was non-significant in multivariable analysis of the one that examined only medical radiation students, potentially due to small sample sizes [28]. The other study was multidisciplinary and found that students with less than 20 cumulative days of rural CP were not any more likely to be practicing rurally from those who did no rural CP, while 21–40 days of rural CP had double the likelihood and more than 40 days had 4.5 times the likelihood [41]. A third study similarly used cumulative CP data to compute three variables: total rural CP, total metro CP, and a ratio of rural-to-metro CP, with the aim of simultaneously examining the effect of metropolitan exposure [40]. Broadly, the study found more rural CP is positively associated with rural practice, while metro CP is negative [40]. 

Few studies examined the role of prior rural interest when describing the relationship of rural CP to practice; however, the potential confounding role of self-selection into rural CP or associated surveys has been noted as a limitation in this literature [24], including in a study using NAHGOT data [28]. This is exemplified in results from a multidisciplinary study from Campbell and Moore (2021) [24]. Health professionals who indicated they were ‘already committed’ when asked about rural CP impact on their rural practice had the highest rates of rural practice. They also had the highest mean intention to be in rural work in five years’ time [24]. 

The evidence for rural CP affecting long-term rural practice is limited and, at most, suggestive of an indirect effect through early-career rural practice. In a follow-up study, Playford et al. (2020) [39] found that having previously lived rurally was no longer significantly associated with rural practice 15–17 years after graduation. Instead, the only significantly positive variable was the rural location of the respondents’ first job after graduation. Unlike in Playford et al. (2006) [38], multivariate analyses in this later study found only rural background to be significant, with the effect of ‘excellent’ placements only approaching significance at the 5% alpha level [39]. However, it should be noted that the follow-up study also included an indicator of whether the student was considering future rural practice at the end of their graduate year, which had the largest coefficient but was only approaching significance. Another multidisciplinary study that found a positive effect for rural CP on rural practice also found that years since graduation was inversely associated with rural practice; however, an interaction effect of the two was not examined [40]. 

Findings from dentistry are mixed on the long-term effect of a voluntary rural CP, with rural CP students significantly more likely to be working rurally than non-CP participants at the initial follow-up 2–6 years later, but not at a second follow-up after an additional two years [33]. However, it was students with positive pre-CP rural practice intentions and ‘prior rural experience’ who were significantly more likely to be working rurally at both touch points [33] (pp. 186–187). Thus, those from a rural background and prior interest in working rurally are more likely to be in the rural workforce in the long term [24,33]. While there is evidence around rural CP which can support early rural practice uptake, this effect appears to diminish with time.

Additionally, there is evidence that rural CP can have both a negative and positive effect on students’ practice location choice. Among occupational therapy professionals in a qualitative study [21], a prior rural CP attracted some of the rural-practising graduates towards rural practice, while the non-rural-practising graduates reported a dissuading effect. Additionally, a study of dentists found those who indicated a rural CP influenced their work location were significantly more likely to be working rurally; however, descriptive statistics also showed 50% of these respondents were working in a metropolitan area [34] (p. 219). Similarly, in a study of allied health graduates, one-third of those who reported their rural CP was influential in their graduate practice location were employed in city locations [23]. 

#### 3.4.3. Use of Theoretical Frameworks to Inform Study Design

Low numbers of papers used a theoretical framework to inform their study. Most papers (*n* = 26, 90%) did not situate their research with a discernible theoretical framework. Three papers (10%) engaged with the following theories or concepts: situated learning *n* = 3 [27,44,45], experiential learning *n* = 2 [44,45], and place-based social processes *n* = 1 [45]. A qualitative paper also identified ‘social capital’ as a theme describing the importance of students’ CP relationships to their supervisor, colleagues, and community for a positive CP experience [36].

## 4. Discussion

This review included twenty-nine studies that examined rural CP in non-medical health professions and their influence upon intention to practice rurally and future rural practice. The main findings of the systematic review are that rural CPs:

(1) Are an avenue for reinforcing or transforming students’ views of rural practice, though this may not necessarily be positive,
(2) When high-quality, can positively influence students to undertake rural practice early in their career, although evidence is limited by self-selection of students into rural training experiences, and
(3) Have scarcely been examined through explicit engagement with theoretical frameworks to inform study methodology. Situated learning theory was the most common theoretical framework employed.

The review found that rural CP experiences can influence students’ intentions or attitudes regarding rural practice, as well as their early-career practice locations. This effect is, however, inconsistently found and appears contingent on student background and prior interest and CP quality. Rural CPs can positively reinforce or increase rural practice intention, particularly among students who are undecided or who do not come from a rural background [36,45,47]. Quality evidence for a positive CP effect on rural practice intention or employment was found in studies where students had rated the rural CP as being of high quality [25], or as providing ‘excellent’ professional development opportunities [38]. There is also some evidence that those who self-select into rural CP are more likely to rate their experience favourably and to report feeling encouraged towards rural practice [25]. However, rural CPs are highly varied experiences, and there was mixed quality evidence indicating that rural CP can negatively influence students’ rural practice intentions [37] and be associated with non-rural practice location choice [21,23,34]. Overall, while those from a rural area are most likely to practice rurally, rural CP experiences are an opportunity to reinforce the values of working rurally, as well as to attract those who are undecided, or whose rural living and working conditions have not yet been a consideration.

The review found no evidence for a positive effect of a rural CP experience on rural practice in the long term, partly attributable to few and only recently commenced longitudinal study designs. It is outside the scope of this review to examine non-CP-related factors in long-term rural workforce commitment; however, one reviewed study of nursing students’ graduate practice intentions reported that exposure to negative aspects of rural practice during CP dissuaded some students from rural practice [37]. Multidisciplinary research indicated that rural CP experiences may support early uptake of rural practice [38,40,41,46], with only early rural practice associated with long-term rural practice outcomes [39], and with likelihood of practising in rural areas being significantly negatively associated with years from graduation [40]. This suggests rural CP may have a ‘foot-in-the-door’ effect [45] for rural employment but not as a sufficient driver in individuals’ decision to stay rural in the long term [44]. See Cosgrave (2020) [49] for further discussion of this issue.

Contrary to policy expectations, this review found high-quality evidence varied on whether longer-term placements foster greater interest in, or realisation of, rural practice for non-medicine health professionals [24,25,28,38,40,41,46]. This may be attributable to the influence of CP quality being more easily examined through student self-reports, highlighting the importance of more rigorous quantitative studies for informing best practice on CP length. 

Several studies did find that high cumulative rural CP length is positively associated with early-career rural practice [24,40,41,46]. Despite this, some were wary of a focus solely on long-term CP given practical constraints. For example, Campbell and Farthing [24] found longer placements to be positively associated with remote practice. They subsequently recommended, given the “increasing competitiveness in securing clinical placements”, that longer and immersive CP should be offered in the final year, while early years of study should look to incorporate shorter and high-quality rural experiences [24] p. 955. While high-quality long-term rural CPs are supported through substantial funding in Australia [2], the volume of CPs required for the large number of non-medicine health students in Australia means students are also likely to experience CPs outside of quality placement models [6,7], which may contribute to a dissuading influence away from rural practice [37]. These mixed findings add further weight to national and international calls for more disciplinary- and context-specific work to understand the positive and negative mechanisms at play in rural CP experiences [1,7,8,50]. The available evidence suggests that those interested in increasing the rural health workforce should focus on high-quality rural CP experiences, including enhancing rural CP more broadly. What ‘quality’ rural CP means, however, requires greater conceptual attention to support meaningful evaluation [50]. Evaluation must also explicitly connect experiences of quality to the impetus of the associated CP model and funding [50].

The overall methodological quality of the included studies was found to be mixed, consistent with a recent multidisciplinary scoping review [8]. Application of the MMAT to assess study quality across different attributes combined with the narrative review found positive study findings were often over-generalised, with methodological limitations largely inadequately addressed. Additionally, controlling for rural background [38] or prior rural experience and pre-CP interest in rural practises appears key for mitigating potential selection bias, which can lead to over-estimation of positive rural CP effects, given that students are more likely to choose a rural placement or end up practising rurally [26,32,38]. A further source of selection bias may be from students with existing rural interest who may be more likely to participate in studies focused on rural placements and provide positive feedback related to these CPs.

While longitudinal studies are promising, the reviewed longitudinal studies were conducted with small samples that inhibit generalisation [33,34], do not have a control group for comparison [39], or do not take into account placement preferences and self-selection into rural CP [40,41,46]. Conversely, when examining change in rural practice interest, findings from this review indicate that ceiling effects may occur among students who have high pre-CP interest in rural practice [47], masking the reinforcing potential of rural CPs. Systematically accounting for these factors in quantitative designs will provide more accurate estimates of rural CP effects on intentions and employment, particularly for assessing the influence of placement length.

Finally, this review found limited engagement with theoretical frameworks among included studies, consistent with critiques on engagement with theory in health education research [10]. Theoretical models support critical interrogation of existing assumptions, expectations, and researcher bias prior to analysis which would enhance methodological rigor [10,11]. ‘Social capital’ has been identified as a key factor in students’ rural CP experience [36], while several studies have described how rural CP may not be an influential factor in practice location decision-making because of other interests and constraints, including existing familial, social, and cultural ties [29,36,44,45]. This indicates there is unrealised potential for social theories to be utilised for informing methodologies and enhancing current understandings of effective mechanisms and contexts for rural CP to affect future practice. Specifically, situated learning theory and practice-based learning frameworks could be used to inform CP design to meet students’ professional development needs and best promote rural practice interest (see: [6,9,11,13]). These approaches are commonly employed in health education research [10,11], and could be incorporated into recent critical work examining place-based understandings of rural communities, health access, and workforce, as well as social accountability frameworks that have informed rural-based health education partnerships (for example [12,14,49]).

### Limitations

The findings of this review were limited by the focus on empirical studies of rural CP for non-medicine health professions students in the Australian context. It excluded studies where medical students were inseparable from other health students on the key measures; however, this was deemed appropriate for the Australian context where rural medical study is distinctly resourced from other health disciplines. This approach, however, means the review encompasses a broad spectrum of non-medicine health disciplines where curricula, placement opportunities, and student characteristics are likely to differ. 

As noted, this study has used adapted assessment criteria for quantitative non-randomised studies to best fit the characteristics of the studies within scope. Although the authors have taken all reasonable steps to apply the MMAT tool appropriately, it is acknowledged that different tools or assessors may yield different assessments of quality.

## 5. Conclusions

This study found that high-quality Australian rural CP experiences can have a positive effect on rural practice intentions and early practice choices for non-medicine health professionals. The reviewed evidence indicated that providing professional development opportunities on rural placements that students view as meaningful and relevant to their practice is important for rural practice intention and early-career employment. Rural CP can also have a dissuading effect due to issues such a social and cultural isolation and poor resourcing of the CP and health services. There were mixed findings on the value of longer rural CP duration. This indicates that those seeking to promote the rural health workforce should focus on lifting the quality of practice experiences overall. The evidence base is currently limited by methodological factors such as varied measures of rural practice intention, potential selection bias, questionable interpretations of statistical analyses, limited long-term data, and low engagement with educational or social theory. There is capacity and need to better inform best-practice policy and CP design for supporting the rural health workforce.

## Figures and Tables

**Table 1:** Article inclusion and exclusion criteria.

Inclusion Criteria	Exclusion Criteria
Students in tertiary-level health professions degrees including medical radiation science, occupational therapy, dentistry, speech pathology/therapy, physiotherapy, nursing, pharmacy, nutrition and dietetics, podiatry, social work, oral health, audiology, orthotics and prosthetics, midwifery, paramedics, psychology, optometry, chiropractic, exercise physiology, and other health courses	Sample included medical or non-health students, where practice intention or employment results were not separable for non-medicine health students
Australian rural, regional, or remote CP studied	Research was not about CP experiences (e.g., simulation, community visits)
	CP were not defined as rural, regional, or remote. CP location was outside of Australia
Report outcomes included rural practice intentions (including ‘interest’ or ‘attractiveness’), and/or rural employment directly attributed to a rural, regional, or remote CP experience	Outcomes were not specific to rural CP. Other outcomes examined only (e.g., placement enjoyment, course progress)
Peer reviewed journal articles of all original study designs	Literature reviews and theses, grey literature, text, and opinion papers
Published between 2000 and 2022	Papers published outside the stated publication range
English language papers	Papers in languages other than English

**Table 2:** Disciplines of health placements included in the literature.

Discipline	Total Number of Studies
Occupational therapy	14
Dentistry	12
Physiotherapy	11
Medical radiation science (incl. radiography, nuclear science, radiation therapy, medical imaging)	10
Nursing	10
Pharmacy	10
Nutrition and dietetics	9
Speech pathology/therapy	9
Podiatry	7
Oral health	5
Social work	5
Audiology	4
Midwifery	4
Paramedicine	4
Psychology	4
Chiropractic	2
Exercise physiology	2
Other health courses	8

**Table 3:** Article characteristics and results of quality screening.

Studies	Duration of Rural CP	Disciplines of CP	Study Design	Report Quality	
				MMAT Rating	MMAT Criteria Met
A mixed-method study of chiropractic student clinical immersion placements in nonmetropolitan Western Australia: influence on student experience, professional attributes, and practice destination [19]	1–2 weeks	Chiropractic	Mixed methods	Low	None
An innovation in Australian dental education: rural, remote, and Indigenous pre-graduation placements [20]	3 weeks	Dentistry	Quantitative descriptive	Low	4.3
Four years after graduation: occupational therapists’ work destinations and perceptions of preparedness for practice [21]	NR	Occupational therapy	Mixed methods	Medium	5.2, 5.3, 5.4
Longitudinal tracking of workplace outcomes for undergraduate allied health students undertaking placements in rural Australia [22]	Short-term CP (<8 weeks), medium-term (8 to 18 weeks), or long-term (semester long or full year) CP	Diagnostic radiography, nuclear science, nutrition and dietetics, occupational therapy, physiotherapy, radiation therapy, and speech pathology	Mixed methods	Low	5.1
Preparing graduates to meet the allied health workforce needs in rural Australia: short-term outcomes from a longitudinal study [23]	Short-term (<8 weeks), medium-term (8 to 18 weeks), or long-term (semester long or full year) CP	Diagnostic radiography, nuclear science, nutrition and dietetics, occupational therapy, physiotherapy, and speech pathology	Mixed methods	Low	5.1
Workplace locations of allied health and nursing graduates who undertook a placement in the Northern Territory of Australia from 2016 to 2019: an observational cohort study [24]	Shorter (2–10 weeks), or longer (10–47 weeks) CP	Nursing, midwifery, audiology, dentistry/oral health, dietetics/nutrition, disability, medical imaging, occupational therapy, optometry, orthotics and prosthetics, paramedicine, pharmacy, physiotherapy, podiatry, psychology, radiation science, social work, speech pathology, and other	Quantitative non-randomized	High	3.1, 3.3, 3.4, 3.5 ^#^
Characteristics of nursing and allied health student placements in the Northern Territory over time (2017–2019) and placement satisfaction [25]	1 ≤ 2 weeks, >2–4 weeks, >4–12 weeks, >12 weeks	Nursing, midwifery, audiology, dentistry, oral health, dietetics/nutrition, disability, medical imaging, occupational therapy, optometry, orthotics and prosthetics, paramedicine, pharmacy, physiotherapy, podiatry, psychology, radiation science, social work, speech pathology, and other	Quantitative non-randomized	High	3.2, 3.3, 3.4, 3.5
The impact of rural clinical placement on student nurses’ employment intentions [26]	4 weeks	Nursing	Quantitative non-randomized	Low	3.1, 3.3 ^#^
Rural placements in Tasmania: do experiential placements and background influence undergraduate health science student’s attitudes toward rural practice? [27]	NR	Nursing, pharmacy, audiology, nutrition and dietetics, occupational therapy, podiatry, physiotherapy, speech therapy, prosthetics, and social work (also medicine).	Quantitative descriptive	Low	4.1, 4.2
Factors influencing medical radiation science graduates’ early-career principal place of practice: a retrospective cohort study [28]	0 days, 1–25 days, 26–50 days, 51+ days	Medical radiation science	Quantitative non-randomized	High	All
Student opinions on a rural placement program in New South Wales, Australia [29]	1 month	Dentistry	Qualitative	Low	None
Assessment of a dental rural teaching program [30]	1 month	Dentistry	Quantitative non-randomized	Low	3.1, 3.3 ^#^
The influence of a clinical rural placement program on the work location of new dental graduates from the University of Sydney, NSW, Australia [31]	1 month	Dentistry	Quantitative non-randomized	Medium	3.1, 3.2, 3.3 ^#^
A longitudinal evaluation of the Rural Clinical Placement Program at the University of Sydney Dental School [32]	1 month	Dentistry	Mixed methods	Low	5.4
A longitudinal workforce analysis of a Rural Clinical Placement Program for final-year dental students [33]	1 month	Dentistry	Quantitative non-randomized	High	All ^#^
The workforce outcomes of dental graduates from a metropolitan school ‘Rural Clinical Placement Program’ versus a ‘Rural Clinical School’ [34]	1 month	Dentistry	Mixed methods	Low	5.1
Pharmacy students’ rural career intentions: perspectives on rural background and placements [35]	2 or 12 weeks	Pharmacy	Mixed methods	Low	None
What do dental students value about their rural placements—Is clinical experience enough? [36]	5 weeks	Dentistry and oral health	Qualitative	Medium	1.3, 1.4, 1.5
The lure of the bush: do rural placements influence student nurses to seek employment in rural settings? [37]	NR	Nursing	Mixed methods	Low	5.4
Going country: rural student placement factors associated with future rural employment in nursing and allied health [38]	>2 weeks	Dietetics, environmental health, health information management, health promotion, medical imaging, nursing, occupational therapy, occupational health and safety, physiotherapy, podiatry, social work, and speech therapy	Quantitative non-randomized	High	3.1, 3.2, 3.4, 3.5 ^#^
Factors associated with rural work for nursing and allied health graduates 15–17 years after an undergraduate rural placement through the University Department of Rural Health program [39]	2–18 weeks	Dietetics, environmental health, health promotion, health information management, health promotion, medical imaging, nursing, occupational therapy, occupational health and safety, pharmacy, physiotherapy, podiatry, social work, and speech therapy	Quantitative non-randomized	High	All ^#^
Does undertaking rural placements add to place of origin as a predictor of where health graduates work? [40]	Duration NR: cumulative days and number of placements calculated, ratio to metro placement days	Dentistry, midwifery, nursing, oral health, occupational therapy, paramedicine, pharmacy physiotherapy, podiatry, and psychology. Nursing analyzed separately.	Quantitative non-randomized	High	3.1, 3.2, 3.3, 3.4 ^#^
Destinations of nursing and allied health graduates from two Australian universities: a data linkage study to inform rural placement models. [41]	Cumulative placement days (0 days, 20 days or less, 21–40 days, more than 40 days) and number of placements calculated	Nursing, occupational therapy, pharmacy, physiotherapy, and medical radiation science	Quantitative non-randomized	High	All ^#^
Pharmacy students’ intention to practice in a rural setting: measuring the impact of a rural curriculum, rural campus, and rural placement on a predominantly metropolitan student cohort [42]	2 or 12 weeks	Pharmacy	Quantitative non-randomized	High	All
Rural pharmacy workforce: influence of curriculum and clinical placement on pharmacists’ choice of rural practice [43]	NR	Pharmacy	Mixed methods	Low	5.1
Up close and real: living and learning in a remote community builds students’ cultural capabilities and understanding of health disparities [44]	2–5 weeks	Speech pathology, occupational therapy, social work, exercise physiology, and generalist health science	Qualitative	Medium	1.3, 1.4 1.5
Learning from follow-up of student placements in a remote community: a small qualitative study highlights personal and workforce benefits and opportunities [45]	3–5 weeks	Occupational therapy, speech pathology, and generalist health science	Qualitative	Medium	1.1, 1.3, 1.4
Rural placements during undergraduate training promote future rural work by nurses, midwives, and allied health professionals [46]	Zero weeks of rural CP compared to 19.4 (nursing)/20.6 (allied health) weeks or greater	Nursing and midwifery, and allied health (analyzed separately) comprising physiotherapy, occupational therapy, social work, speech pathology, dietetics, pharmacy, exercise physiology, psychology, paramedicine, podiatry, radiography, medical laboratory science, audiology, radiation therapy, sonography, optometry, dentistry, oral health, other allied health	Quantitative non-randomized	Low	3.4, 3.4 ^#^
Immersive placement experiences promote rural intent in allied health students of urban and rural origin [47]	1 week to 12 months	Medical radiation science, nutrition and dietetics, occupational therapy, physiotherapy, and speech pathology	Mixed methods	High	5.1, 5.2, 5.3, 5.4

^#^ Using adapted MMAT criterion 3.5, ‘Is the statistical analysis appropriate to answer the research question?’. CP: clinical placement, MMAT: Mixed Methods Appraisal Tool, NR: not reported.

**Table 4:** Review findings presented by outcome (intention and/or employment) studied.

Studies	Theory or Conceptual Framework	Rural Background/Interest Incl. in Analysis	Intention and/or Employment Examined	Outcome Indicator	Results
A mixed-method study of chiropractic student clinical immersion placements in nonmetropolitan Western Australia: Influence on student experience, professional attributes, and practice destination [19]	NR	No	Intention and employment	More likely to consider practicing in a rural or remote setting as a result of CP and rurality of employment locations	Graduates who were working in a rural location were more likely to have voluntarily undertaken a rural CP during their degree.
An innovation in Australian dental education: rural, remote, and Indigenous pre-graduation placements [20]	NR	No	Intention	Consideration of rural practice	Most students reported considering rural practice prior to the CP at similar levels to post-CP, despite most not having experienced rural living before placement.
Four years after graduation: occupational therapists’ work destinations and perceptions of preparedness for practice [21]	NR	No	Employment	Described influence of rural CP experiences on rural or metro employment location	Rural CP enticed some of the rural-practicing graduates towards rural practice, while all seven of the non-rural-practicing graduates reported their rural CP had a dissuading effect.
Longitudinal tracking of workplace outcomes for undergraduate allied health students undertaking placements in Rural Australia [22]	NR	Yes	Intention	Pre- and post-CP rural work intentions	“38.3% positive change” between allied health students’ retrospective self-assessment of rural practice intention pre-CP to their post-CP rating, with 55% of students with no rural background having a higher rating.
Preparing graduates to meet the allied health workforce needs in rural Australia: short-term outcomes from a longitudinal study [23]	NR	Yes	Employment	Influence of CP on current rural or metro employment, employment location one year after graduation	Students from a non-rural background were significantly more likely to have indicated that their CP influenced their decision and be practicing rurally. No significant difference was found among rural students. One-third of allied health students who indicated that their CP experience influenced them to take up their graduate position were employed in a city location.
Workplace locations of allied health and nursing graduates who undertook a placement in the Northern Territory of Australia from 2016 to 2019: an observational cohort study [24]	NR	Yes	Employment	Whether employed in a rural area ^, whether rural placement influenced consideration of rural practice, intention to work as a rural or remote health professional within the first 5 years following graduation (0 = no intention; 50 = 50/50 probability; 100 = absolute certainty)	Graduates who spent more than 10 weeks on CP were more likely to be working rurally. Three-quarters of respondents reported that placement influenced their consideration of rural practice and were more likely to be practicing rurally. Those who indicated they were ‘already committed’ to rural practice had highest rates of rural practice and highest mean intention of being in rural work in five years’ time.
Characteristics of nursing and allied health student placements in the Northern Territory over time (2017–2019) and placement satisfaction [25]	NR	Partially. Prior consideration of rural work examined	Intention	Placement has encouraged consideration of living and working in a rural or remote location	Those who reported prior consideration of rural living and working were more likely to be encouraged by CP, as were those who reported satisfaction with educational resources and overall. Rural background and placement length not significant in univariate tests.
The impact of rural clinical placement on student nurses’ employment intentions [26]	NR	Partially	Intention	Intention to seek work in a rural area	Proportion of students intending to practice rurally was significantly higher among those who chose a rural CP than among those who chose a metro CP. This difference in intention between groups was significantly higher both pre- and post-CP. No significant change in the proportion of students intending to practice rurally post-CP.
Rural placements in Tasmania: do experiential placements and background influence undergraduate health science student’s attitudes toward rural practice? [27]	Situated learning	Yes (but not separated by discipline)	Intention	Pre- and post-CP rural work intention ratings	Participants of all disciplines areas and geographical backgrounds increased their mean rural work intention ratings. This was significant in all groups except pharmacy and rural classification areas, which had sample sizes of seven or less
Factors influencing medical radiation science graduates’ early-career principal place of practice: a retrospective cohort study [28]	NR	Yes	Employment	Rural employment 2 years after graduation	Multivariate analysis found rural background the sole predictor of rural practice; neither number of CP nor cumulative CP days were significant beyond univariate models.
Student opinions on a rural placement program in New South Wales, Australia [29]	NR	Partially	Intention	Intention to work rurally, pre- and post-CP, description of how CP raised interest	Factors of the rural CP experience reported to raise interest in rural practice, including positive experiences with the rural community and patients, a broader range of clinical procedures, a shorter commute to work, quality supervision, and clinical mentors.
Assessment of a dental rural teaching program [30]	NR	Yes	Intention	Intention to work rurally, pre- and post-CP	55% of pre-CP participants were interested in working rurally after they graduate with the rest undecided. All post-CP participants, apart from one, were interested (97%).
The influence of a clinical rural placement programme on the work location of new dental graduates from the University of Sydney, NSW, Australia [31]	NR	No	Employment	Rural employment location two and three years after graduation	Participants in the voluntary rural CP were significantly more likely to be practicing rurally.
A longitudinal evaluation of the Rural Clinical Placement Program at the University of Sydney Dental School [32]	NR	Partially	Employment	Rural employment location, whether rural CP had a positive influence on employment location	Participants in the voluntary rural CP had higher likelihood of being employed rurally at follow-up, approaching significance at the 5% alpha level. Respondents who agreed their rural CP experience influenced their working location were more likely to be working rurally.
A longitudinal workforce analysis of a Rural Clinical Placement Program for final year dental students [33]	NR	Yes	Employment	Rural employment location at two time points	Participants in the voluntary rural CP were significantly more likely to be working rurally than non-participants at initial follow-up but not at the second follow-up two years later, although these graduates were significantly more likely to be retained rurally between years.
The workforce outcomes of dental graduates from a metropolitan school ‘Rural Clinical Placement Program’ versus a ‘Rural Clinical School’ [34]	NR	Yes (for quantitative component only)	Employment	Rural employment location, positive influence of rural CP on employment location	Participants in the voluntary rural CP had higher likelihood of being employed in rural practice, approaching significance at the 5% alpha level. Graduates who indicated that the rural CP influenced their work location were significantly more likely to be working rurally; 50% of those who responded that it did impact their work location were working in a metropolitan area.
Pharmacy students’ rural career intentions: perspectives on rural background and placements [35]	NR	Yes	Intention	Rating whether rural CP increased likelihood of working in a rural area	High agreeance from students who have undertaken at least one rural CP reporting it as a valuable learning experience and made them more likely to work in a rural area. No difference by whether they had a rural background or not.
What do dental students value about their rural placements—is clinical experience enough? [36]	NR	Partially (predominately metro sample)	Intention	Interest in rural practice post-placement	Most would consider rural living and working after CP predominately because of the practice opportunities, with responses focused on their positive CP experience. Those who did not described social connections to city and city lifestyle as reasons.
The lure of the bush: do rural placements influence student nurses to seek employment in rural settings? [37]	NR	Partially	Intention	Whether would consider working rurally, whether would consider working rurally on graduation	Rural CP gave students “good insight” on what a graduate year might look like in a rural hospital, although this could have both negative and positive impacts on practice considerations. No clear numbers reported.
Going country: rural student placement factors associated with future rural employment in nursing and allied health [38]	NR	Yes	Employment	Rural employment one year after graduation	The two CP factors that were significantly, positively associated with future rural practice when controlling for rural background were where the CP was rated by students as ‘excellent’ for their professional development as well as those whose rural CP was for four weeks or less.
Factors associated with rural work for nursing and allied health graduates 15-17 years after an undergraduate rural placement through the University Department of Rural Health program [39]	NR	Yes	Employment	Rural employment at one and 15–17 years after graduation	No rural CP characteristics significantly associated with long-term rural practice, only whether the first job after graduation was in a rural location. Rural background also non-significant.
Does undertaking rural placements add to place of origin as a predictor of where health graduates work? [40]	NR	Yes	Employment	Employment in metropolitan, regional, rural, or remote areas in early years of practice	Higher ratio of metro to rural CP significantly, negatively associated with rurality of practice. Rural background was significant and positive, accounting for the greatest amount of variance in rural practice.
Destinations of nursing and allied health graduates from two Australian universities: a data linkage study to inform rural placement models [41]	NR	Yes	Employment	Rural practice in second year post-graduation	0–20 cumulative days of placement—not significantly different from zero rural CP, 21–40 days—double likelihood of rural practice than zero CP, more than 40 days—associated with 4.5 times the likelihood of rural CP with the rural background indicator returning similarly high odds.
Pharmacy students’ intention to practise in a rural setting: measuring the impact of a rural curriculum, rural campus and rural placement on a predominantly metropolitan student cohort [42]	NR	Yes	Intention	Intention to practice rurally	Rural CP is positively associated with rural practice intention but is only approaching significance. Rural background is the only significant factor found.
Rural pharmacy workforce: influence of curriculum and clinical placement on pharmacists’ choice of rural practice [43]	NR	Yes	Employment	Choice to practice rurally, how placement influenced this choice	Pharmacists reported their rural CP experiences as “predominately positively influencing their choice of rural career” (p. 134), with the opportunity to experience a rural lifestyle indicated as a significant influence by most.
Up close and real: living and learning in a remote community builds students’ cultural capabilities and understanding of health disparities [44]	Interprofessional learning, experiential and situated learning	Partially	Intention and employment	Graduate feedback on CP impact, rural employment proportion	Placement ‘reaffirmed’ students’ existing interest in practicing rurally. Six of eight employed recent graduates were working rurally. One student interested in working remotely in the long-term.
Learning from follow-up of student placements in a remote community: a small qualitative study highlights personal and workforce benefits and opportunities [45]	Experiential and situated learning frameworks, place-based social processes	Yes	Intention	Graduates’ retrospective assessment of CP influence on their rural practice intentions	Rural CP experience was perceived to provide substantial professional development for students which positively influenced or reinforced existing positive attitudes.
Rural placements during undergraduate training promote future rural work by nurses, midwives, and allied health professionals [46]	NR	Yes	Employment	Hours worked in rural practice within the last week (1–14 years after graduation)	Health professionals who reported the highest quintile of rural CP weeks during their studies reported working significantly longer hours in rural practice than those who undertook no placement.
Immersive placement experiences promote rural intent in allied health students of urban and rural origin [47]	NR	Yes	Intention	Cross-sectional before and after CP rural work intention ratings	Intention rated higher for both rural background and non-rural background students, but only significantly higher among non-rural students. Significantly higher ratings were found for all CP lengths and disciplines, except for medical radiation which had existing high ratings.

NR: not reported. ^ classified as medium rural town to very remote; excludes larger rural towns and regional centers.

## Data Availability

Not applicable.

## Appendix A

Exemplar of search strategy.

(AB ((rural*) OR (regional*) OR (remote))) AND (AB ((clinical placement) OR (student placement))) AND (AB ((health*) OR (medic*) OR (nurs*) OR (clinical) OR (social work))) AND (AB ((recruitment) OR (retention) OR (intention*) OR (workforce) OR (employment) OR (career))).

## References

[1] WHO Increasing Access to Health Workers in Remote and Rural Areas through Improved Retention: Global Policy Recommendations. https://apps.who.int/iris/handle/10665/44369.

[2] Lyle D., Greenhill J. (2018). Two decades of building capacity in rural health education, training and research in Australia: University Departments of Rural Health and Rural Clinical Schools. Aust. J. Rural Health.

[3] Holst J. (2020). Increasing Rural Recruitment and Retention through Rural Exposure during Undergraduate Training: An Integrative Review. Int. J. Environ. Res. Public Health.

[4] Walsh S., Lyle D.M., Thompson S.C., Versace V.L., Browne L.J., Knight S., Pit S.W., Jones M. (2020). The role of national policies to address rural allied health, nursing and dentistry workforce maldistribution. Med. J. Aust..

[5] Jackson D. (2016). Re-conceptualising graduate employability: The importance of pre-professional identity. High. Educ. Res. Dev..

[6] Billett S., Kennedy M., Billett S., Gherardi S., Grealish L. (2015). The practices of using and integrating practice-based learning in higher education. Practice-Based Learning in Higher Education.

[7] Battye K., Sefton C., Thomas J.M., Smith J., Springer S., Skinner I., Callander E., Butler S., Wilkins R., Gordon J. (2020). Independent Evaluation of the Rural Health Multidisciplinary Training Program: Final Report to the Commonwealth Department of Health.

[8] Moran A., Nancarrow S., Cosgrave C., Griffith A., Memery R. (2020). What works, why and how? A scoping review and logic model of rural clinical placements for allied health students. BMC Health Serv. Res..

[9] McGrath C., Liljedahl M., Palmgren P.J. (2019). You say it, we say it, but how do we use it? Communities of practice: A critical analysis. Med. Educ..

[10] Rees C.E., Monrouxe L.V. (2010). Theory in medical education research: How do we get there?. Med. Educ..

[11] O’Brien B.C., Battista A. (2020). Situated learning theory in health professions education research: A scoping review. Adv. Health Sci. Educ..

[12] Malatzky C., Bourke L. (2016). Re-producing rural health: Challenging dominant discourses and the manifestation of power. J. Rural Stud..

[13] Roberts C., Daly M., Held F., Lyle D. (2017). Social learning in a longitudinal integrated clinical placement. Adv. Health Sci. Educ..

[14] Roberts P., Cosgrave C., Gillespie J., Malatzky C., Hyde S., Hu W.C., Bailey J., Yassine T., Downes N. (2021). ‘Re-placing’ professional practice. Aust. J. Rural. Health.

[15] Page M.J., McKenzie J.E., Bossuyt P.M., Boutron I., Hoffmann T.C., Mulrow C.D., Shamseer L., Tetzlaff J.M., Akl E.A., Brennan S.E. (2021). The PRISMA 2020 statement: An updated guideline for reporting systematic reviews. BMJ.

[16] Beks H., Walsh S., Alston L., Jones M., Smith T., Maybery D., Sutton K., Versace V.L. (2022). Approaches Used to Describe, Measure, and Analyze Place of Practice in Dentistry, Medical, Nursing, and Allied Health Rural Graduate Workforce Research in Australia: A Systematic Scoping Review. Int. J. Environ. Res. Public Health.

[17] Australian Bureau of Statistics (ABS) (2018). 1270.0.55.005-Australian Statistical Geography Standard (ASGS): Volume 5—Remoteness Structure, July 2016. https://www.abs.gov.au/AUSSTATS/abs@.nsf/Lookup/1270.0.55.005Main+Features1July%202016?OpenDocument.

[18] Hong Q.N., Fàbregues S., Bartlett G., Boardman F., Cargo M., Dagenais P., Gagnon M.-P., Griffiths F., Nicolau B., O’Cathain A. (2018). The Mixed Methods Appraisal Tool (MMAT) version 2018 for information professionals and researchers. Educ. Inf..

[19] Amorin-Woods L.G., Losco B.E., Leach M.J. (2019). A mixed-method study of chiropractic student clinical immersion placements in nonmetropolitan Western Australia: Influence on student experience, professional attributes, and practice destination. J. Chiropr. Educ..

[20] Bazen J., Kruger E., Dyson K., Tennant M. (2007). An innovation in Australian dental education: Rural, remote and Indigenous pre-graduation placements. Rural Remote Health.

[21] Brockwell D., Wielandt T., Clark M. (2009). Four years after graduation: Occupational therapists’ work destinations and perceptions of preparedness for practice. Aust. J. Rural Health.

[22] Brown L., Smith T., Wakely L., Wolfgang R., Little A., Burrows J. (2017). Longitudinal Tracking of Workplace Outcomes for Undergraduate Allied Health Students Undertaking Placements in Rural Australia. J. Allied Health.

[23] Brown L., Smith T., Wakely L., Little A., Wolfgang R., Burrows J. (2017). Preparing Graduates to Meet the Allied Health Workforce Needs in Rural Australia: Short-Term Outcomes from a Longitudinal Study. Educ. Sci..

[24] Campbell N., Farthing A., Lenthall S., Moore L., Anderson J., Witt S., Rissel C. (2021). Workplace locations of allied health and nursing graduates who undertook a placement in the Northern Territory of Australia from 2016 to 2019: An observational cohort study. Aust. J. Rural Health.

[25] Campbell N., Moore L., Farthing A., Anderson J., Witt S., Lenthall S., Petrovic E., Lyons C., Rissel C. (2021). Characteristics of nursing and allied health student placements in the Northern Territory over time (2017–2019) and placement satisfaction. Aust. J. Rural Health.

[26] Courtney M., Edwards H., Smith S., Finlayson K. (2002). The impact of rural clinical placement On Student Nurses’ Employment Intentions. Collegian.

[27] Dalton L.M., Routley G.K., Peek K.J. (2008). Rural placements in Tasmania: Do experiential placements and background influence undergraduate health science student’s attitudes toward rural practice?. Rural. Remote Health.

[28] Farrugia L., Smith T., Depczynski J. (2021). Factors influencing medical radiation science graduates’ early career principal place of practice: A retrospective cohort study. J. Med. Radiat. Sci..

[29] Johnson G.E., Blinkhorn A.S. (2011). Student opinions on a rural placement program in New South Wales, Australia. Rural Remote Health.

[30] Johnson G., Blinkhorn A. (2012). Assessment of a dental rural teaching program. Eur. J. Dent..

[31] Johnson G., Blinkhorn A. (2013). The influence of a clinical rural placement programme on the work location of new dental graduates from the U niversity of S ydney, NSW, A ustralia. Eur. J. Dent. Educ..

[32] Johnson G., Wright F.A.C., Foster K. (2019). A longitudinal evaluation of the Rural Clinical Placement Program at the University of Sydney Dental School. Eur. J. Dent. Educ..

[33] Johnson G., Byun R., Foster K., Wright F., Blinkhorn A., Wright F.A.C. (2019). A longitudinal workforce analysis of a Rural Clinical Placement Program for final year dental students. Aust. Dent. J..

[34] Johnson G., Blinkhorn A., Byun R., Foster K., Wright F.A.C. (2020). The workforce outcomes of dental graduates from a metropolitan school ‘Rural Clinical Placement Program’ versus a ‘Rural Clinical School’. Int. Dent. J..

[35] Kirschbaum M., Khalil H., Talyor S., Page A.T. (2016). Pharmacy students’ rural career intentions: Perspectives on rural background and placements. Curr. Pharm. Teach. Learn..

[36] Koedyk C., Satur J., Vaughan B. (2021). What do dental students value about their rural placements—Is clinical experience enough?. Aust. J. Rural Health.

[37] Lea J., Cruickshank M., Paliadelis P., Parmenter G., Sanderson H., Thornberry P. (2008). The lure of the bush: Do rural placements influence student nurses to seek employment in rural settings?. Collegian.

[38] Playford D., Larson A., Wheatland B. (2006). Going country: Rural student placement factors associated with future rural employment in nursing and allied health. Aust. J. Rural Health.

[39] Playford D., Moran M.C., Thompson S. (2020). Factors associated with rural work for nursing and allied health graduates 15-17 years after an undergraduate rural placement through the University Department of Rural Health program. Rural Remote Health.

[40] Skinner T.C., Semmens L., Versace V., Bish M., Skinner I.K. (2021). Does undertaking rural placements add to place of origin as a predictor of where health graduates work?. Aust. J. Rural. Health.

[41] Sutton K., Depczynski J., Smith T., Mitchell E., Wakely L., Brown L.J., Waller S., Drumm D., Versace V.L., Fisher K. (2021). Destinations of nursing and allied health graduates from two Australian universities: A data linkage study to inform rural placement models. Aust. J. Rural Health.

[42] Taylor S.J., Maharaj P., Williams K., Sheldrake C. (2009). Pharmacy students’ intention to practise in a rural setting: Measuring the impact of a rural curriculum, rural campus and rural placement on a predominantly metropolitan student cohort. Aust. J. Rural Health.

[43] Taylor S.M., Lindsay D., Glass B.D. (2019). Rural pharmacy workforce: Influence of curriculum and clinical placement on pharmacists’ choice of rural practice. Aust. J. Rural Health.

[44] Thackrah R.D., Hall M., Fitzgerald K., Thompson S.C. (2017). Up close and real: Living and learning in a remote community builds students’ cultural capabilities and understanding of health disparities. Int. J. Equity Health.

[45] Thackrah R.D., Thompson S.C. (2019). Learning from follow-up of student placements in a remote community: A small qualitative study highlights personal and workforce benefits and opportunities. BMC Med. Educ..

[46] Thomas J., Butler S., Battye K., Sefton C., Smith J., Skinner I., Springer S., Callander E. (2021). Rural placements during undergraduate training promote future rural work by nurses, midwives and allied health professionals. Aust. J. Rural Health.

[47] Wolfgang R., Wakely L., Smith T., Burrows J., Little A., Brown L.J. (2019). Immersive placement experiences promote rural intent in allied health students of urban and rural origin. J. Multidiscip. Health.

[48] Sutton K.P., Beauchamp A., Smith T., Waller S., Brown L., Fisher K., Woodfield M., Major L., Depczynski J., Versace V.L. (2021). Rationale and protocol for the Nursing and Allied Health Graduate Outcomes Tracking (NAHGOT) study: A large-scale longitudinal investigation of graduate practice destinations. Rural Remote Health.

[49] Cosgrave C. (2020). The Whole-of-Person Retention Improvement Framework: A Guide for Addressing Health Workforce Challenges in the Rural Context. Int. J. Environ. Res. Public Health.

[50] Green E., Quilliam C., Sheepway L., Hays C.A., Moore L., Rasiah R.L., Bailie J., Howard C., Hyde S., Inyang I. (2022). Identifying features of quality in rural placements for health students: Scoping review. BMJ Open.

